# Whole-Genome Sequencing Analysis of Salmonella enterica Serovar Enteritidis Isolates in Chile Provides Insights into Possible Transmission between Gulls, Poultry, and Humans

**DOI:** 10.1128/AEM.01760-16

**Published:** 2016-09-30

**Authors:** Magaly Toro, Patricio Retamal, Sherry Ayers, Marlen Barreto, Marc Allard, Eric W. Brown, Narjol Gonzalez-Escalona

**Affiliations:** a Instituto de Nutrición y Tecnología de los Alimentos, Universidad de Chile, Macul, Santiago, Chile; b Facultad de Ciencias Veterinarias y Pecuarias, Universidad de Chile, La Pintana, Santiago, Chile; c U.S. Food and Drug Administration, Center for Veterinary Medicine, Laurel, Maryland, USA; d Center of Biomedical Research, Faculty of Health Sciences, Universidad Autónoma de Chile, Santiago, Chile; e U.S. Food and Drug Administration, Center for Food Safety and Applied Nutrition, College Park, Maryland, USA; Pennsylvania State University

## Abstract

Salmonella enterica subsp. enterica serotype Enteritidis is a major cause of human salmonellosis worldwide; however, little is known about the genetic relationships between *S*. Enteritidis clinical strains and *S*. Enteritidis strains from other sources in Chile. We compared the whole genomes of 30 *S*. Enteritidis strains isolated from gulls, domestic chicken eggs, and humans in Chile, to investigate their phylogenetic relationships and to establish their relatedness to international strains. Core genome multilocus sequence typing (cgMLST) analysis showed that only 246/4,065 shared loci differed among these Chilean strains, separating them into two clusters (I and II), with cluster II being further divided into five subclusters. One subcluster (subcluster 2) contained strains from all surveyed sources that differed at 1 to 18 loci (of 4,065 loci) with 1 to 18 single-nucleotide polymorphisms (SNPs), suggesting interspecies transmission of *S*. Enteritidis in Chile. Moreover, clusters were formed by strains that were distant geographically, which could imply that gulls might be spreading the pathogen throughout the country. Our cgMLST analysis, using other *S*. Enteritidis genomes available in the National Center for Biotechnology Information (NCBI) database, showed that *S*. Enteritidis strains from Chile and the United States belonged to different lineages, which suggests that *S*. Enteritidis regional markers might exist and could be used for trace-back investigations.

**IMPORTANCE** This study highlights the importance of gulls in the spread of Salmonella Enteritidis in Chile. We revealed a close genetic relationship between some human and gull *S*. Enteritidis strains (with as few as 2 of 4,065 genes being different), and we also found that gull strains were present in clusters formed by strains isolated from other sources or distant locations. Together with previously published evidence, this suggests that gulls might be spreading this pathogen between different regions in Chile and that some of those strains have been transmitted to humans. Moreover, we discovered that Chilean *S*. Enteritidis strains clustered separately from most of *S*. Enteritidis strains isolated throughout the world (in the GenBank database) and thus it might be possible to distinguish the geographical origins of strains based on specific genomic features. This could be useful for trace-back investigations of foodborne illnesses throughout the world.

## INTRODUCTION

*S*almonella enterica subsp. enterica serotype Enteritidis is one of the most prevalent S. enterica serotypes in Chile and throughout the world (1, 2). This pathogen is considered an emerging foodborne zoonosis, and evidence shows a clear epidemiological association between commercial poultry products (mainly undercooked eggs and meat) and human disease (1, 3–5). In Chile, *S*. Enteritidis emerged as a pathogen of public health importance in 1994, and both the poultry industry and public health services maintain active surveillance for this pathogen (1). Recently, studies have suggested that *S*. Enteritidis in Chile could potentially be transmitted among wild birds, domesticated poultry, and humans; this could partially explain the recent increased prevalence of human salmonellosis in Chile (6, 7). However, that assessment was derived by combining the discriminative powers of pulsed-field gel electrophoresis (PFGE), virulence types, and phenotypic characteristics (7), which might be ineffective for *S*. Enteritidis because it has very low genetic diversity (3, 8). Although PFGE is the gold standard for foodborne outbreak investigations, it lacks sufficient resolution to determine whether two *S*. Enteritidis isolates are phylogenetically related (8). In the present study, we wanted to investigate, through genomic analyses, whether *S*. Enteritidis strains isolated from different sources in Chile are phylogenetically related and whether strains isolated from gulls indeed pose a risk to the health of humans or domesticated animals.

Investigating these questions also will allow us to explore growing evidence that some S. enterica serotypes possess biogeographical structures. For example, the whole-genome sequences of S. enterica serotype Newport strains isolated in Asia cluster separately from those of *S*. Newport isolates from other parts of the world (9). More recently, a combination of whole-genome sequencing (WGS) and geographical metadata allowed tracing of S. enterica Bareilly strains from a foodborne outbreak in the United States back to sites in India (10). Some *S*. Enteritidis lineages can be linked to geographical locations such as Africa or California, suggesting that migratory fauna could have a role in spreading some *S*. Enteritidis lineages to other parts of the world (3). Allard et al. demonstrated that WGS had the ability to distinguish *S*. Enteritidis strains from two farms producing contaminated eggs (8), and two other recent U.S. outbreaks were identified in New York and Minnesota (11, 12); therefore, it is reasonable to expect that WGS of *S*. Enteritidis will be useful for distinguishing isolates across a wider geographical area. While evidence indicates that some lineages and clades of *S*. Enteritidis could be linked closely to geographical locations, whether *S*. Enteritidis strains isolated in Chile can be reliably differentiated from *S*. Enteritidis strains isolated from other parts of the globe has not been established.

To address these questions, we sequenced the genomes of 30 *S*. Enteritidis strains isolated from different hosts in Chile and conducted WGS analysis by core genome multilocus sequence typing (cgMLST) and maximum likelihood (ML) analysis of the single-nucleotide polymorphisms (SNPs) identified in the core genome loci. cgMLST analysis has previously been used for tracking and surveillance of Mycobacterium tuberculosis (13), methicillin-resistant Staphylococcus aureus (MRSA) (14), Listeria monocytogenes (15), Neisseria (16), and Vibrio parahaemolyticus (17). Our aims were to identify phylogenetic relationships among *S*. Enteritidis strains isolated from different hosts and to compare their genomic sequences with those of strains from other geographical regions.

## MATERIALS AND METHODS

### Bacterial strains.

*S*. Enteritidis strains (*n* = 30) isolated from gulls (*n* = 10; fecal samples), chicken eggs (*n* = 9; donated by María Esther Saldías, Servicio Agrícola y Ganadero, Chile), and humans (*n* = 11; donated by Alda Fernández, Instituto de Salud Publica, Chile) were collected between 2009 and 2012 from locations around Chile (Table 1) and were stored in Microbank cryogenic vials at −80°C in the Laboratory of Infectious Diseases, Veterinary Medicine School, University of Chile. Strains were confirmed as Salmonella spp. with a Vitek 2 system (bioMérieux, Durham, NC).

**TABLE 1:** Salmonella enterica serotype Enteritidis strains isolated from different sources in Chile

Strain	CFSAN^*a*^ no.	Year of isolation	Region of isolation^*b*^	Sample type	Source (if known)	Alias	GenBank accession no.
SAL4628	CFSAN024726	2009	Valparaíso	Food	Egg (chicken)	SEN2	LIIZ00000000
SAL4629	CFSAN024727	2009	Valparaíso	Food	Egg (chicken)	SEN5	LILV00000000
SAL4630	CFSAN024728	2009	Valparaíso	Food	Egg (chicken)	SEN7	LILU00000000
SAL4631	CFSAN024729	2010	Antofagasta	Clinical	Human	SEN21	LILT00000000
SAL4632	CFSAN024730	2010	RM	Clinical	Human	SEN22	LILS00000000
SAL4633	CFSAN024731	2011	Arica	Food	Egg (chicken)	SEN31	LILR00000000
SAL4634	CFSAN024732	2012	Arica	Food	Egg (chicken)	SEN47	LILQ00000000
SAL4635	CFSAN024733	2012	Atacama	Clinical	Human	SEN49	LILP00000000
SAL4636	CFSAN024734	2012	Valparaíso	Clinical	Human	SEN53	LILO00000000
SAL4637	CFSAN024735	2012	RM	Clinical	Human	SEN63	LILN00000000
SAL4638	CFSAN024736	2012	Valparaíso	Clinical	Human	SEN71	LIMD00000000
SAL4639	CFSAN024737	2012	Coquimbo	Clinical	Human	SEN72	LIMC00000000
SAL4640	CFSAN024738	2012	Magallanes	Clinical	Human	SEN73	LIMB00000000
SAL4641	CFSAN024739	2012	Valparaíso	Clinical	Human	SEN77	LIMA00000000
SAL4642	CFSAN024740	2012	RM	Clinical	Human	SEN78	LILZ00000000
SAL4643	CFSAN024741	2012	RM	Clinical	Human	SEN79	LILY00000000
SAL4644	CFSAN024742	2012	Valdivia	Food	Egg (chicken)	SEN82	LILX00000000
SAL4645	CFSAN024743	2012	RM	Food	Egg (chicken)	SEN87	LILW00000000
SAL4646	CFSAN024744	2012	RM	Food	Egg (chicken)	SEN88	LIOP00000000
SAL4647	CFSAN024745	2012	RM	Food	Egg (chicken)	SEN89	LIOQ00000000
SAL4648	CFSAN024746	2011	Arica	Fecal	Franklin gull	SEN95	LIOR00000000
SAL4649	CFSAN024747	2012	Coquimbo	Fecal	Kelp gull	SEN97	LIOS00000000
SAL4650	CFSAN024748	2012	Coquimbo	Fecal	Kelp gull	SEN98	LIOZ00000000
SAL4651	CFSAN024749	2012	Coquimbo	Fecal	Kelp gull	SEN99	LIOY00000000
SAL4652	CFSAN024750	2012	Coquimbo	Fecal	Kelp gull	SEN101	LIPA00000000
SAL4653	CFSAN024751	2012	Coquimbo	Fecal	Kelp gull	SEN107	LIOX00000000
SAL4654	CFSAN024752	2012	Valparaíso	Fecal	Kelp gull	SEN110	LIOW00000000
SAL4655	CFSAN024753	2012	Valparaíso	Fecal	Kelp gull	SEN111	LIOV00000000
SAL4656	CFSAN024754	2012	Valparaíso	Fecal	Kelp gull	SEN112	LIOU00000000
SAL4657	CFSAN024755	2012	Valparaíso	Fecal	Kelp gull	SEN127	LIOT00000000

a CFSAN, Center for Food Safety and Applied Nutrition.

b RM, Region Metropolitana de Santiago (capital area).

### DNA extraction.

Strains were grown overnight at 37°C in tryptic soy broth (TSB), and then DNA was extracted from individual samples using a DNeasy blood and tissue kit (Qiagen, Valencia, CA). DNA concentrations were measured with a Qubit fluorometer (Life Technologies, Carlsbad, CA) and standardized to 0.2 ng/μl, and samples were stored at −20°C prior to library preparation.

### Library preparation and sequencing.

Libraries were prepared with a Nextera XT DNA sample preparation kit (Illumina, San Diego, CA), according to the manufacturer's instructions. Genomes were sequenced using Illumina MiSeq sequencing technology, with 500 (2 × 250) cycles and a pair-end library with coverage depth of 60- to 190-fold, at the FDA Center for Food Safety and Applied Nutrition genomics laboratory.

### Genomic data analysis.

Whole-genome sequence contigs for each strain were *de novo* assembled using CLC Genomics Workbench v7.5.1 (Qiagen, Germantown, MD). The CLC *de novo* assembly algorithm uses de Bruijn graphs to represent overlapping reads. The imported reads were trimmed by quality (quality scores of >30), and Nextera adapters were removed. To analyze the relationships among our *S*. Enteritidis Chilean strains, we performed *in silico* multilocus sequence typing (MLST) and cgMLST using Ridom Seqsphere+ v2.3 (Ridom GmbH, Germany). The cgMLST analysis using Ridom Seqsphere first defined a cgMLST scheme using the cgMLST target definer tool with default settings within the software. The genome of *S*. Enteritidis strain P125109 (GenBank accession no. NC_011294) was used as the reference genome (4,160 genes) (18). Then the genome of *S*. Enteritidis strain EC20121175 (GenBank accession no. CP007269.2) was used for comparison with the reference genome to establish a list of core and accessory genome genes. Genes that were repeated in more than one copy in either of the two genomes were removed from the analysis as failed genes. After this, a task template containing both core and accessory genes was created for future testing. Each individual locus (core or accessory gene) was designated allele number 1. Then assemblies for each individual *S*. Enteritidis genome were queried against the task template; if the locus was found and was different from the reference genome or any other queried genome, then a new number was assigned to that locus. cgMLST performed a gene-by-gene analysis and identified SNPs within different alleles to establish genetic distance calculations. We then inferred the evolutionary history of the isolates with the ML method, based on the Kimura 2-parameter model (19). All positions with missing data were eliminated. Analyses were conducted in MEGA v6 (20). The statistical support of the nodes in the ML tree was assessed by bootstrap resampling with 1,000 replicates.

### Genomes for comparison.

We used the cgMLST approach described above to compare the phylogeny of Chilean *S*. Enteritidis isolates with that of *S*. Enteritidis isolates from other geographical areas that were archived in GenBank (http://www.ncbi.nlm.nih.gov/genome/genomes/152?genome_assembly_id=group154368). Some *S*. Enteritidis genomes were excluded because of low quality, and 274 genomes were used for the final analysis (see Table S1 in the supplemental material).

### Targeted polymorphism analysis.

During the cgMLST analysis, we used Ridom Seqsphere+ to identify all genes with allelic differences among our strains. Then polymorphisms were characterized for every allele by searching for the specific sequence in the reference genome and verifying the allelic change in each codon. Finally, we used the Virulence Factors of Pathogenic Bacteria database (http://www.mgc.ac.cn/cgi-bin/VFs/genus.cgi?Genus=Salmonella) of the Institute of Pathogen Biology, Chinese Academy of Medical Sciences and Peking Union Medical College (Beijing, China), to identify the predicted pathogenicity genes associated with those polymorphisms.

### Antimicrobial resistance analysis.

We determined phenotypically the antimicrobial MICs of our strains using the Sensititre automated microbial susceptibility testing system (Trek Diagnostic Systems, Westlake, OH), and data were interpreted according to Clinical and Laboratory Standards Institute MIC standards (21). Escherichia coli ATCC 25922, Enterococcus faecalis ATCC 29212, Staphylococcus aureus ATCC 29213, and Pseudomonas aeruginosa ATCC 27853 were used as controls for antimicrobial MIC determinations. We tested the following antimicrobials: azithromycin, amoxicillin-clavulanic acid, ampicillin, cefoxitin, ceftiofur, ceftriaxone, chloramphenicol, ciprofloxacin, gentamicin, nalidixic acid, streptomycin, sulfisoxazole, tetracycline, and trimethoprim-sulfamethoxazole. Additionally, we used ResFinder (Center for Genomic Epidemiology, Technical University of Denmark, Lyngby, Denmark) to detect the presence or absence of acquired antimicrobial resistance genes in the draft genomes (22). We set a 60% identity threshold and a 60% minimum length to search for target genes.

### Accession number(s).

All genomes were submitted to GenBank; accession numbers are presented in Table 1.

## RESULTS

### MLST and cgMLST analyses of Chilean *S*. Enteritidis strains.

Our *in silico* MLST analysis of the 30 Chilean *S*. Enteritidis strains revealed that 28 strains (93%) belonged to sequence type 11 (ST 11) (see Table S1 in the supplemental material), the most common *S*. Enteritidis ST in the Salmonella MLST database (http://mlst.warwick.ac.uk/mlst/dbs/Senterica). For two strains (strains SAL4632 and SAL4650), we discovered a novel ST (ST3000) that differed from ST11 by 1 SNP in the *hemD* locus, at position 278 (T→G, compared to *hemD*3). This transversion in the second codon position results in a nonsynonymous change from L to R, in comparison with the ST11 variant.

**FIG 1:**
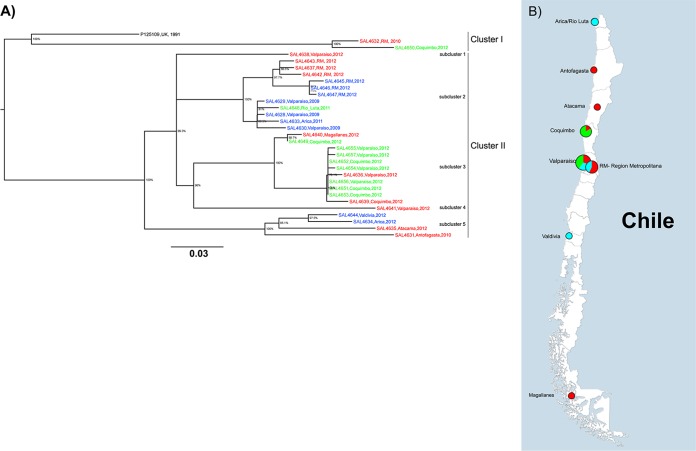
Whole-genome phylogenetic analysis of Salmonella Enteritidis isolated in Chile from different sources, based on cgMLST results. (A) Maximum likelihood tree using the Kimura 2-parameter model (19). The tree is drawn to scale, with branch lengths measured in the number of substitutions per site. All positions containing gaps and missing data were eliminated. Evolutionary analyses were conducted with MEGA v6 (20). Different alleles were found at 246 core genome loci (see Table S3 in the supplemental material). SNPs can be found in Table S4 in the supplemental material. Red, human clinical isolates; green, gull isolates; blue, food (egg) isolates. (B) Map of Chile and locations where *S*. Enteritidis strains were isolated. Colors in the pie charts represent strains isolated in each area, and sizes are proportional to the number of strains isolated in the area. Red, human clinical isolates; green, gull isolates; blue, food (egg) isolates. The map was created with Ridom SeqSphere+ v3.1, as part of the software's geocoding feature.

Using cgMLST and reference genome *S*. Enteritidis strain P125109 (GenBank accession no. NC_011294) (18), we identified a total of 4,160 genes. Our Chilean *S*. Enteritidis strains shared 4,065 of those genes (core genes) (see Table S2 in the supplemental material). Only 246 core genes (6.1%), displaying a total of 281 SNPs, were polymorphic. The ML analysis divided the strains into two clusters (Fig. 1A; also see Tables S3 and S4 in the supplemental material), differing at 55 to 95 loci with 55 to 97 SNPs. Cluster I contained the reference strain P125109 (a clinical strain associated with a poultry outbreak in the United Kingdom) and only two Chilean *S*. Enteritidis strains, i.e., SAL4632 (human) and SAL4650 (kelp gull, Larus dominicanus). They differed among themselves at 12 to 58 loci with 12 to 59 SNPs. These two strains presented MLST profiles different from those of all the other Chilean *S*. Enteritidis strains we studied (Fig. 1A and Table 1), but the two samples were originally isolated 400 km apart. Cluster II included 93% of the Chilean strains (28/30 strains) and was divided into five subclusters (Fig. 1A), differing at 0 to 65 loci with 0 to 65 SNPs. Subclusters 1 and 4 were composed of single human strains (SAL4638 and SAL4641, respectively). Subcluster 2 included 11 strains from all three hosts, differing at 1 to 18 loci with 1 to 18 SNPs. Subcluster 3 was formed by 11 strains isolated from humans and gulls, differing at 0 to 16 loci with 0 to 16 SNPs. Subcluster 5 contained strains isolated from humans and chicken eggs, differing at 8 to 36 loci with 44 to 57 SNPs (Fig. 1A). Subclusters 2, 3, and 5 contained at least one strain isolated as much as 2,500 km from the other strains in the cluster (Fig. 1B).

**FIG 2:**
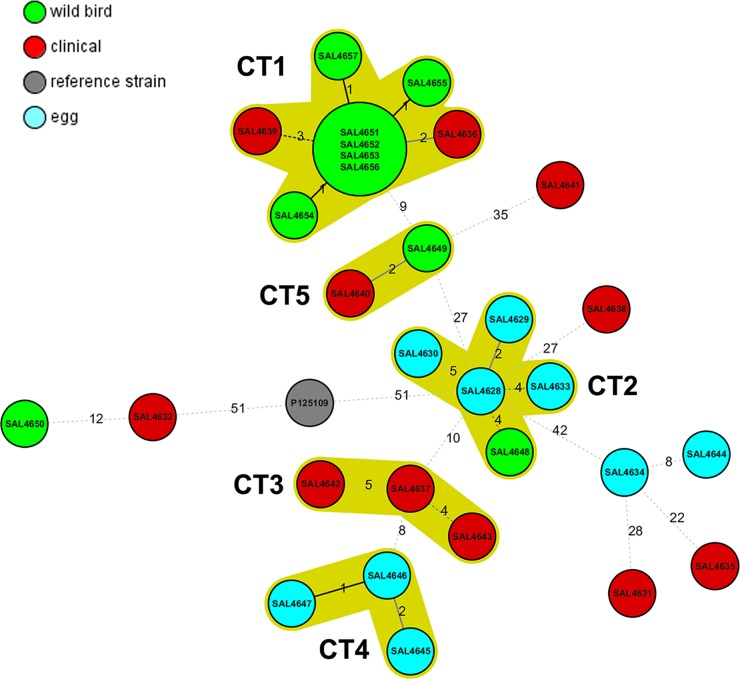
Cluster types found among Salmonella Enteritidis strains isolated from different sources in Chile (2009 to 2012). The minimum spanning tree was based on the same number of loci as in Fig. 1. Numbers by lines, number of loci differing between strains or complexes. Solid lines, strains differing at 1 or 2 loci; dashed lines, strains differing at ≥3 loci. Red, human clinical isolates; green, gull isolates; blue, food (egg) isolates; gray, *S*. Enteritidis strain P125109 (reference genome). Complexes were defined as groups of strains that differed at ≤5 loci. The lines are not drawn to scale.

A minimum spanning tree grouped the strains into five cluster types (CTs) (groups of strains differing at ≤5 alleles). The largest CT (CT1) consisted of nine strains differing at 1 to 4 loci. Four of those isolates (SAL4651, SAL4652, SAL4653, and SAL4656), which had been isolated from kelp gulls sampled in coastal cities located 400 km apart (Valparaíso and Coquimbo), had identical cgMLST profiles (Fig. 2 and Table 1). Two CTs were composed of either human or egg strains (CT3 and CT4, respectively), and the other two CTs contained a variety of strains from gulls and eggs (CT2) or gulls and humans (CT5). Nine strains did not fit into any CT.

### cgMLST of Chilean *S*. Enteritidis strains and other *S*. Enteritidis strains in GenBank.

**FIG 3:**
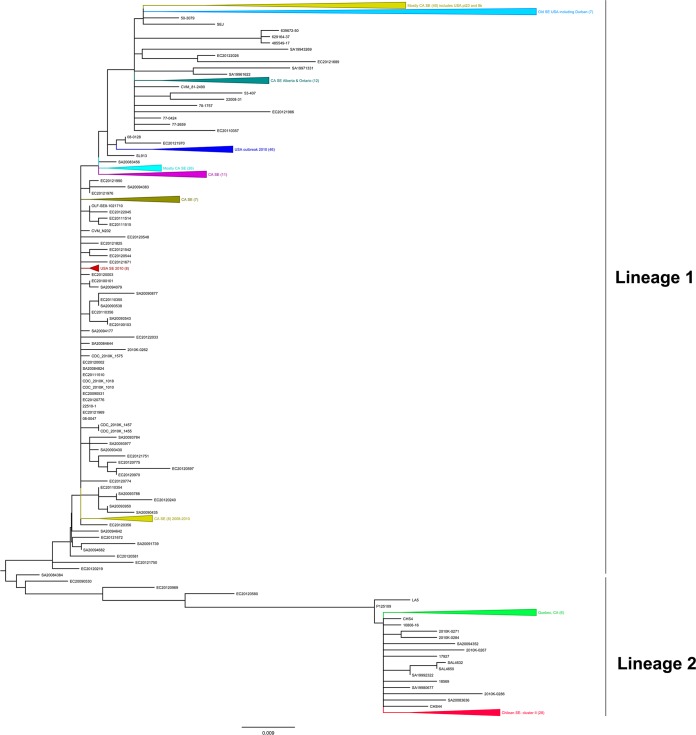
Whole-genome phylogenetic analysis of Salmonella Enteritidis sequences available in GenBank (*n* = 274) and Chilean *S*. Enteritidis isolates (*n* = 30), based on cgMLST results. The neighbor-joining tree was based on a cgMLST analysis that identified 1,171 loci present in every *S*. Enteritidis isolate in GenBank (see Table S5 in the supplemental material). The SNPs can be found in Table S6 in the supplemental material. Triangles, strain clusters. The tree was exported in the Newick format and modified using FigTree v1.4.2. Most of the clusters were collapsed into groups for easy visualization (for the whole tree, see Fig. S1 in the supplemental material). Chilean *S*. Enteritidis strains are represented in red in lineage 2. Lineage 1 contained mostly U.S. and Canadian *S*. Enteritidis strains.

Our cgMLST analysis, using 274 *S*. Enteritidis genomes available from GenBank (see Table S1 in the supplemental material) and 30 *S*. Enteritidis strains from Chile, identified 1,181 genes that were shared by all *S*. Enteritidis genomes (*n* = 304) (see Table S5 in the supplemental material). The SNPs identified are presented in Table S6 in the supplemental material. An ML phylogenetic tree constructed using SNPs from the 1,181 loci from all 304 *S*. Enteritidis genomes divided the *S*. Enteritidis strains into two main lineages (Fig. 3). Lineage I included *S*. Enteritidis strains from the United States, Canada, and China, but the *S*. Enteritidis strains from Chile were located in lineage II. Almost all Chilean strains (28/30 strains) were grouped together, except for 2 that were still in the same lineage, clustering nearby. Lineage II also included strain P125109 (GenBank accession no. NC_011294), the *S*. Enteritidis type strain used as the genome reference for our cgMLST analysis. Of the 51 strains in lineage II, 2 strains were from China (strains CHS4 and CHS44), 2 were from the United Kingdom (strains LA5 and P125109), 1 was from the United States (strain 17927), 1 was from Mexico (strain 18569), 10 were from Canada, and 30 were from Chile; 5 were of unknown origin (Fig. 3; also see Table S4 in the supplemental material).

### Targeted polymorphism analysis.

Among the Chilean strains studied, we detected 281 polymorphisms among 246 core genes, represented by 7 nonsense (2.5%), 78 silent (27.8%), and 178 missense (63.3%) mutations. All of the nonsense mutations were found in human clinical strains, and 5 of them, involving structural and metabolic coding sequences, were found in the same 2 strains, i.e., SAL4632 and SAL4650 (Table 2). Importantly, we identified mutations in 12 predicted virulence genes for *S*. Enteritidis (Table 3).

**TABLE 2:** Pseudogenes (nonsense mutations) detected in Chilean Salmonella enterica serovar Enteritidis isolates

Gene location^*a*^	Gene/protein name	Strain(s) involved	Description
SEN1363	Invasin	SAL4632 (human), SAL4650 (wild bird)	Invasin-like protein
SEN2039	*pduD*	SAL4632 (human), SAL4650 (wild bird)	Diol dehydratase medium subunit
SEN2609	Hypothetical protein	SAL4641 (human)	Type I secretion system predicted effector
SEN2795	*steB*	SAL4638 (human)	Outer membrane usher protein; fimbrial adherence determinant
SEN2861	*kduL*	SAL4632 (human), SAL4650 (wild bird)	4-Deoxy-l-threo-5-hexosulose-uronate ketol-isomerase
SEN3758	*yigM*	SAL4632 (human), SAL4650 (wild bird)	Hypothetical membrane protein
SEN4207	*mgtA*	SAL4632 (human), SAL4650 (wild bird), P125109 (reference strain)	Magnesium transport

a Gene location, location in *S*. Enteritidis strain P125109 (reference genome in the cgMLST analysis).

**TABLE 3:** Predicted virulence genes with SNPs in Chilean Salmonella Enteritidis

Gene location	Gene name	Description^*a*^
SEN0528	*fimH*	FimH protein; fimbrial adherence determinant
SEN0952	*pipB*	SPI2 protein; type III secretion system-translocated effector
SEN1628	SSAp gene	Type III secretion protein; type III secretion system needle length regulator
SEN1825	*sifA*	SPI2 protein; type III secretion system-translocated effector; required for formation of lysosomal glycoprotein
SEN2065	*sopA*	SPI1 protein; type III secretion system-translocated effector
SEN2717	*hilD*	SPI1 protein; type III secretion system transcriptional regulator; AraC family
SEN2784	*sopD*	SPI1 hypothetical protein; type III secretion system-translocated effector
SEN2795	*steB*^*b*^	Outer membrane usher protein; fimbrial adherence determinant
SEN3463	*lpfA*	Long polar fimbrial protein A; fimbrial adherence determinant
SEN3941	*sseK1*	SPI2 hypothetical protein; type III secretion system-translocated effector, non-LEE-encoded effector protein NleB
SEN4248	*sefB*	Fimbrial chaperone protein; fimbrial adherence determinant
SEN4347	*sthE*	Fimbrial subunit; fimbrial adherence determinant

a SPI, Salmonella pathogenicity island; LEE, locus of enterocyte effacement.

b Nonsense mutation.

### *In vitro* and *in silico* assessments of antimicrobial resistance.

Our *in vitro* analyses determined that 8 gull, 2 chicken egg, and 3 human clinical isolates exhibited antimicrobial resistance (Table 4). The most common phenotype was simultaneous resistance to streptomycin and tetracycline (11/13 isolates); all strains displaying this phenotype were in subcluster 2. One poultry egg strain, SAL4630, showed multidrug resistance to nalidixic acid, ciprofloxacin, tetracycline, and trimethoprim-sulfamethoxazole. We did not find any Chilean strains resistant to the other nine antimicrobials (Table 4).

**TABLE 4:** Phenotypic antimicrobial susceptibility testing results for Salmonella strains from Chile

Strain	Source	Testing result^*a*^					No. of drug resistances per strain
		NAL	STR	CIP	TET	COT	
SAL4629	Poultry	R	S	R	S	S	2
SAL4630	Poultry	R	S	R	R	R	4
SAL4636	Human	S	R	S	R	S	2
SAL4639	Human	S	R	S	R	S	2
SAL4640	Human	S	R	S	R	S	2
SAL4649	Kelp gull	S	R	S	R	S	2
SAL4651	Kelp gull	S	R	S	R	S	2
SAL4652	Kelp gull	S	R	S	R	S	2
SAL4653	Kelp gull	S	R	S	R	S	2
SAL4654	Kelp gull	S	S	S	R	S	1
SAL4655	Kelp gull	S	R	S	R	S	2
SAL4656	Kelp gull	S	R	S	R	S	2
SAL4657	Kelp gull	S	R	S	R	S	2

a Breakpoints were adopted from Clinical and Laboratory Standards Institute guidelines (21). All 30 *S*. Enteritidis strains were susceptible to amoxicillin-clavulanic acid, ampicillin, azithromycin, cefoxitin, ceftiofur, ceftriaxone, chloramphenicol, sulfisoxazole, and gentamicin. Only resistant strains are presented. S, sensitive; R, resistant; NAL, nalidixic acid (resistance at ≥32 mg/liter); STR, streptomycin (resistance at ≥64 mg/liter) (no CLSI interpretive criteria for this bacterium-antimicrobial combination are currently available); CIP, ciprofloxacin (resistance at ≥1 mg/liter); TET, tetracycline (resistance at ≥16 mg/liter); COT, trimethoprim-sulfamethoxazole (resistance at ≥4/76 mg/liter). The numbers of resistant strains were as follows: nalidixic acid, 2/30 strains; streptomycin, 10/30 strains; ciprofloxacin, 2/30 strains; tetracycline, 12/30 strains; trimethoprim-sulfamethoxazole, 1/30 strains.

Our *in silico* analyses used ResFinder (https://cge.cbs.dtu.dk/services/ResFinder) to detect acquired antimicrobial resistance genes located either in plasmids or in the chromosome, usually as part of integrons. These results agreed with those obtained from the phenotypic analysis; no antimicrobial resistance genes were found in strains that had not shown antimicrobial resistance patterns *in vitro*. Gene *qnrB19* (plasmid) was identified in two strains (SAL4629 and SAL4630) that were resistant to nalidixic acid, *strA*, *strB*, and *tetA* (plasmid) were identified in strains that were resistant to streptomycin and tetracycline (*n* = 10) (Table 5), and *sulI* (plasmid) and *dfrA25* (integron) were found in a strain (SAL4630) that displayed resistance to sulfisoxazole and trimethoprim-sulfamethoxazole (Table 5).

**TABLE 5:** *In silico* antimicrobial resistance profiles of Salmonella Enteritidis strains isolated in Chile

Strain	Source	Region of isolation	Resistance gene(s) detected^*a*^
SAL4629	Poultry	Valparaíso	*qnrB19*
SAL4630	Poultry	Valparaíso	*qnrB19*, *sul1*, *tet*(A), *dfrA25*
SAL4636	Human	Valparaíso	*strA*, *strB*, *tet*(A)
SAL4639	Human	Coquimbo	*strB*, *strA*, *tet*(A)
SAL4640	Human	Magallanes	*strB*, *strA*, *tet*(A)
SAL4649	Kelp gull	Coquimbo	*strB*, *strA*, *tet*(A)
SAL4651	Kelp gull	Coquimbo	*strB*, *strA*, *tet*(A)
SAL4652	Kelp gull	Coquimbo	*strB*, *strA*, *tet*(A)
SAL4653	Kelp gull	Coquimbo	*strB*, *strA*, *tet*(A)
SAL4654	Kelp gull	Valparaíso	*strB*, *strA*, *tet*(A)
SAL4655	Kelp gull	Valparaíso	*strB*, *strA*, *tet*(A)
SAL4656	Kelp gull	Valparaíso	*strB*, *strA*
SAL4657	Kelp gull	Valparaíso	*strB*, *strA*, *tet*(A)

a *qnrB19*, transferable quinolone resistance determinant; *sul1*, sulfonamide-resistant dihydropteroate synthase that cannot be inhibited by sulfonamide; *tet*(A), major facilitator superfamily transporter, tetracycline efflux pump; *dfrA25*, group A drug-insensitive dihydrofolate reductase that cannot be inhibited by trimethoprim; *strA*, streptomycin resistance; *strB*, streptomycin resistance.

## DISCUSSION

Chilean *S*. Enteritidis strains isolated from different sources exhibit very similar genetic profiles, independent of their sources and hosts. In fact, identifying Chilean strains from each host (gulls, eggs, and humans) in the same subcluster in our maximum likelihood phylogenetic tree suggests that some strains are already circulating among different hosts and food vehicles in Chile. These findings fit with prior evidence of similar molecular profiles among *S*. Enteritidis strains collected from humans and chickens (22) and from humans and other animals (23). Our analyses also identified a close relationship between *S*. Enteritidis strains isolated from humans and gulls, indicating that birds might represent an epidemiologically relevant reservoir of zoonotic *S*. Enteritidis in Chile (Fig. 1A). Similarly, Bell et al. recently reported close relationships among *S*. Newport strains isolated from geese, freshwater, and clinical patients in the United States (24), underlining the importance of wild birds in zoonotic salmonellosis throughout the world.

Our comparisons between the Chilean *S*. Enteritidis genomes and *S*. Enteritidis sequences from around the world that were available in GenBank demonstrated that all Chilean *S*. Enteritidis strains clustered in lineage II, well separated from the 2010 U.S. egg outbreak strains and from most of the *S*. Enteritidis sequences available in GenBank. Although *S*. Enteritidis lineage II also contains *S*. Enteritidis strains from Canada and China and the *S*. Enteritidis reference strain P125109 (18) from the United Kingdom (Fig. 3), we have shown that Chilean *S*. Enteritidis genomes are more similar among themselves than similar to other *S*. Enteritidis strains isolated elsewhere in the world, indicating some geographically restricted subpopulations of *S*. Enteritidis.

Salmonella Enteritidis is a highly clonal serotype within Salmonellae (8). In our study, we found that 3,819/4,065 genes (94.0%) were identical across *S*. Enteritidis Chilean strains, with a low percentage of polymorphisms in core genes, i.e., 246/4,065 genes (6.1%), confirming a low level of diversity. Among 281 SNPs, 12 were detected in predicted pathogenicity genes, including a nonsense mutation in the *steB* gene (a fimbrial adherence determinant) from a clinical strain (Tables 2 and 3). Pseudogenization of these sequences suggests that complementary functions are encoded in the genome to support adaptation to different host environments. Experimental evidence suggested a minor role for *mgtA* in the *in vivo* virulence of Salmonella (25), possibly explaining its apparent loss of function through negative selection. However, this process might also result from progressive changes in lifestyle, e.g., from free-living to facultative intracellular organism, a process in which some gene products can yield deleterious functions (26, 27). Whatever the cause, pseudogenization increases bacterial adaptation to the environment, although the specific effects of these mutations in Chilean *S*. Enteritidis strains still need to be determined. The majority of the nonsense mutations were found in human clinical strains, with most (5/7 mutations) being observed in strains SAL4632 (human) and SAL4650 (gull) (26, 27). Our phylogenetic study revealed that these two *S*. Enteritidis strains appeared more closely related to each other and to reference strain P125109 (United Kingdom) (18) than to any other Chilean *S*. Enteritidis strains in our study (Fig. 1A), suggesting that strain SAL4650 may be a zoonotic pathogen. Further laboratory studies are necessary to fully characterize this strain.

Most *S*. Enteritidis isolates worldwide belong to ST11 (28), which held true for most of our *S*. Enteritidis isolates. Only two strains (SAL4632 and SAL4650) presented a different ST, differing from the pattern of ST11 by a single, previously undescribed, SNP in the housekeeping gene *hemD* (http://mlst.warwick.ac.uk/mlst/mlst/dbs/Senterica). Strains SAL4632 (human) and SAL4650 (gull) shared not only a mutated *hemD* gene but also other mutations (Table 2). Consequently, our phylogenetic analysis indicated that those two strains differed from other Chilean *S*. Enteritidis strains enough to form a second sublineage (Fig. 1A); those strains did not share the host or geographical origin of the other isolates. Previous studies demonstrated the presence of several sublineages of *S*. Enteritidis in Chile (1, 29). Fica et al. described differences in *S*. Enteritidis phage types from 1990 to 2000, due to the introduction of poultry lines from outside the country (1). In 2003, Fernandez et al. analyzed 441 Chilean *S*. Enteritidis strains isolated between 1975 and 1993, and they described the presence of two *S*. Enteritidis groups, with two subclusters each (29). Later, Rios et al. classified Chilean *S*. Enteritidis isolates collected between 2001 and 2003 into four subgroups (30). While they detected 13 *S*. Enteritidis subtypes, 88% of the strains clustered together, forming one large subcluster (30). At the moment, no other Chilean *S*. Enteritidis genome sequences are available to confirm the presence of additional sublineages. Although we found two well-defined Chilean *S*. Enteritidis sublineages in this study, we analyzed only a limited number of strains; more clusters may emerge if we sequence a broader range of Chilean *S*. Enteritidis strains.

In the Chilean population, several animal food commodities, including eggs, meat, fish, and shellfish (among others), have been involved in *S*. Enteritidis outbreaks, suggesting several transmission chains among bacteria and hosts. Recent studies have shown potential relationships between wild bird *S*. Enteritidis isolates and human *S*. Enteritidis strains in Chile, which may explain the increased incidence of salmonellosis in the country (6, 7). Unpublished data from the Institute of Public Health (the Chilean national reference laboratory for Salmonella) suggest that water runoffs from poultry and livestock industries could explain the contamination of bodies of water that ultimately affects coastal environments, where multidrug-resistant bacteria have been detected in gulls (7). Therefore, Salmonella in water represents an indirect transmission pathway that allows dissemination to different animal hosts (domestic and wild, terrestrial and marine) and ultimately to derived food commodities, perhaps perpetuating the transmission cycle. Our data reveal that as few as two or three genes differ among strains isolated from gulls and humans, confirming that those strains can potentially infect humans. Strain SAL4648 (Rio Luta/gull) is the only gull strain in a cluster formed by *S*. Enteritidis strains from the three sources studied. That strain differs by only four alleles from egg strain SAL4628 (Valparaíso) (Fig. 2); interestingly, those strains were isolated over 2,000 km apart (Fig. 1B). It is unclear how those very similar strains came to be present in chicken eggs and gulls from different regions.

In previous studies, Salmonella spp. displayed geographical structures in the world (9, 10). We detected two clusters and five subclusters formed by Chilean *S*. Enteritidis strains isolated from different hosts. Each multistrain subcluster displayed some geographical restriction, that is, strains clustered together were isolated from nearby areas (Fig. 1A and B). In each one of those groups, however, there was at least one strain isolated as much as 1,500 km from the others in the subcluster. As mentioned previously, cluster I was formed by two strains separated by over 400 km (Valparaíso and Coquimbo). Subcluster 2 (cluster II) was composed of strains from the metropolitan region and Arica (1,663 km apart). Subcluster 3 strains were isolated in the Valparaíso/Coquimbo area and Magallanes (2,585 km apart), and subcluster 5 was formed by strains isolated from the north of the country (Arica, Antofagasta, and Atacama) and Valdivia (2,392 km apart). Globally, we also observed that Chilean *S*. Enteritidis strains clustered together with some Canadian *S*. Enteritidis strains (Fig. 3). A probable explanation for this phenomenon is that migratory birds might be vehicles for *S*. Enteritidis strains along the Chilean coast and probably along continents. Data from other studies documenting the phylogenetic relatedness of influenza viruses detected in Chilean seabirds (including kelp gulls) and North American wild birds (31) suggest that the migratory patterns of the animals could contribute to disease transmission along those routes, which might partially explain the clustering of Chilean strains and some Canadian strains in this work (Fig. 3), as well as the composition of the clusters and subclusters across the country (Fig. 1A and B). Therefore, we think that there is the potential for interspecies transmission of *S*. Enteritidis on a national and international scale. Additional research is needed to identify the direction and routes of potential contamination, and systematic sampling of hosts would be useful for explaining the detection of phylogenetically related bacterial strains over such long distances. An alternative explanation for the isolation of closely related strains from distant locations in Chile could be related to the vertically integrated poultry industry adopted in the country (only a few companies provide poultry products to the entire country, from a few centralized sources), which could spread the same *S*. Enteritidis strains (if present in poultry) over long distances. However, this could not explain the isolation of genetically similar Salmonella Enteritidis strains from gulls.

An important part of properly characterizing the risk that any of these strains might pose to human health is identifying antimicrobial resistance patterns. We did not detect discrepancies between predicted *S*. Enteritidis antimicrobial resistance determined by *in silico* analysis of *S*. Enteritidis genomes and the results of phenotypic testing, confirming the utility of *in silico* methods. Nearly one-half of the strains (13/30 strains) we analyzed displayed acquired antimicrobial resistance; of those strains, 77% were resistant to both streptomycin and tetracycline. Interestingly, most resistant strains (8/13 strains) had been isolated from gulls, with almost all showing the same antimicrobial pattern (7/8 strains). Three human strains showed the same antimicrobial resistance profile. Since the antimicrobial resistance genes identified in Chilean *S*. Enteritidis strains had been identified previously in plasmids (32, 33), we think that such plasmids might have been transmitted across strains inhabiting different hosts. Remarkably, most of the strains displaying antimicrobial resistance clustered together in cluster II, subcluster 3, and exhibited the same *in silico* and *in vitro* antimicrobial resistance gene profiles.

Taken together, the close relationship between human and gull strains, the anthropogenic impact on the environment, and the transmission of antibiotic resistance plasmids across bacteria suggest that there may be a high risk of interspecies transmission of multidrug-resistant *S*. Enteritidis in Chile. The annual report of the Latin American Surveillance Network of Antimicrobial Resistance indicated that *S*. Enteritidis is the second Salmonella serotype in Chile with higher levels of antimicrobial resistance (34). Clinical and environmental strains of *S*. Enteritidis from Chile were resistant to nalidixic acid, ciprofloxacin, co-trimoxazole, and nitrofurantoin, while resistance to ampicillin, amoxicillin, and chloramphenicol was found only in clinical strains and resistance to tetracycline was reported only for strains from poultry or wildlife (34). Because that report did not mention whether isolates were tested for resistance to streptomycin, it is unclear whether resistance to both streptomycin and tetracycline is common among *S*. Enteritidis strains isolated in Chile or Latin America.

Analyzing the genomic contents of strains using cgMLST focuses attention on the genes shared across all of our strains (16, 17, 35) and provides resolution comparable to that of SNP-based WGS typing. We found that cgMLST provided a robust analysis, rapidly revealing phylogenetic relatedness among isolates in a format that permitted easy data exchange and storage (17, 35). Two other notable benefits of cgMLST are that it requires less computational power than whole-genome SNP analysis and it can be performed by users with limited bioinformatics backgrounds; these features make this type of analysis more accessible to the worldwide scientific community.

Our initial hypothesis was that it should be possible to differentiate Chilean *S*. Enteritidis strains from strains originating from different geographical areas by means of WGS analysis. Our results confirmed that Chilean *S*. Enteritidis strains clustered apart from most other *S*. Enteritidis sequences available in GenBank but still close to certain strains isolated from other parts of the world (Fig. 3). The results showed that, despite the confounding effects of worldwide poultry transport, there are two main lineages of *S*. Enteritidis in the world, although a clear defined biogeographical structure for *S*. Enteritidis could not be established. In the case of Chilean *S*. Enteritidis strains, distribution was subject to some geographical restriction. We think that it will eventually be possible to identify unique genetic markers in *S*. Enteritidis that are linked to location, such as certain “hot spot” mutation areas recently revealed in the genome of *S*. Bareilly (10). Identifying such markers will help us design new methods for trace-back investigations of foodborne and zoonotic outbreaks.

In conclusion, our data indicated that *S*. Enteritidis strains isolated in Chile from different hosts were diverse, although phylogenetically related; in some cases, there seemed to be a close relationship between gull and human clinical strains, indicating the potential for interspecies transmission and indicating that gulls could be an important reservoir for human disease in Chile. Moreover, the presence of two different types or sublineages of *S*. Enteritidis in Chile was revealed, with the majority of strains belonging to ST11. Since Chilean strains clustered together in our global *S*. Enteritidis genome comparison, there might be the potential to discover regional markers within their genomes. This study also highlighted the use of whole-genome sequencing for traceability of *S*. Enteritidis strains in Chile and the rest of the world.

## Supplementary Material

Supplemental material

## References

[1] Fica A, Acosta G, Dabanch J, Perret C, Torres M, Lopez J, Jofre L, Weitzel T 2012 Salmonellosis outbreaks and the size and role of the Chilean State. Rev Chilena Infectol 29:207–214. doi:10.4067/S0716-10182012000200014 (In Spanish.) 22689037

[2] Hendriksen RS, Vieira AR, Karlsmose S, Lo Fo Wong DM, Jensen AB, Wegener HC, Aarestrup FM 2011 Global monitoring of Salmonella serovar distribution from the World Health Organization Global Foodborne Infections Network Country Data Bank: results of quality assured laboratories from 2001 to 2007. Foodborne Pathog Dis 8:887–900. doi:10.1089/fpd.2010.0787. 21492021

[3] Deng X, Desai PT, den Bakker HC, Mikoleit M, Tolar B, Trees E, Hendriksen RS, Frye JG, Porwollik S, Weimer BC, Wiedmann M, Weinstock GM, Fields PI, McClelland M 2014 Genomic epidemiology of Salmonella enterica serotype Enteritidis based on population structure of prevalent lineages. Emerg Infect Dis 20:1481–1489. doi:10.3201/eid2009.131095. 25147968 PMC4178404

[4] Cardoen S, Van HX, Berkvens D, Quoilin S, Ducoffre G, Saegerman C, Speybroeck N, Imberechts H, Herman L, Ducatelle R, Dierick K 2009 Evidence-based semiquantitative methodology for prioritization of foodborne zoonoses. Foodborne Pathog Dis 6:1083–1096. doi:10.1089/fpd.2009.0291. 19715429

[5] Jackson BR, Griffin PM, Cole D, Walsh KA, Chai SJ 2013 Outbreak-associated Salmonella enterica serotypes and food commodities, United States, 1998–2008. Emerg Infect Dis 19:1239–1244. doi:10.3201/eid1908.121511. 23876503 PMC3739514

[6] Fresno M, Barrera V, Gornall V, Lillo P, Paredes N, Abalos P, Fernandez A, Retamal P 2013 Identification of diverse Salmonella serotypes, virulotypes, and antimicrobial resistance phenotypes in waterfowl from Chile. Vector Borne Zoonotic Dis 13:884–887. doi:10.1089/vbz.2013.1408. 24107205 PMC3868272

[7] Retamal P, Fresno M, Dougnac C, Gutierrez S, Gornall V, Vidal R, Vernal R, Pujol M, Barreto M, Gonzalez-Acuna D, Abalos P 2015 Genetic and phenotypic evidence of the Salmonella enterica serotype Enteritidis human-animal interface in Chile. Front Microbiol 6:464. doi:10.3389/fmicb.2015.00464. 26029196 PMC4432690

[8] Allard MW, Luo Y, Strain E, Pettengill J, Timme R, Wang C, Li C, Keys CE, Zheng J, Stones R, Wilson MR, Musser SM, Brown EW 2013 On the evolutionary history, population genetics and diversity among isolates of Salmonella Enteritidis PFGE pattern JEGX01.0004. PLoS One 8:e55254. doi:10.1371/journal.pone.0055254. 23383127 PMC3559427

[9] Cao G, Meng J, Strain E, Stones R, Pettengill J, Zhao S, McDermott P, Brown E, Allard M 2013 Phylogenetics and differentiation of Salmonella Newport lineages by whole genome sequencing. PLoS One 8:e55687. doi:10.1371/journal.pone.0055687. 23409020 PMC3569456

[10] Hoffmann M, Luo Y, Monday SR, Gonzalez-Escalona N, Ottesen AR, Muruvanda T, Wang C, Kastanis G, Keys C, Janies D, Senturk IF, Catalyurek UV, Wang H, Hammack TS, Wolfgang WJ, Schoonmaker-Bopp D, Chu A, Myers R, Haendiges J, Evans PS, Meng J, Strain EA, Allard MW, Brown EW 2016 Tracing origins of the Salmonella Bareilly strain causing a food-borne outbreak in the United States. J Infect Dis 213:502–508. doi:10.1093/infdis/jiv297. 25995194

[11] den Bakker HC, Allard MW, Bopp D, Brown EW, Fontana J, Iqbal Z, Kinney A, Limberger R, Musser KA, Shudt M, Strain E, Wiedmann M, Wolfgang WJ 2014 Rapid whole-genome sequencing for surveillance of Salmonella enterica serovar enteritidis. Emerg Infect Dis 20:1306–1314. doi:10.3201/eid2008.131399. 25062035 PMC4111163

[12] Taylor AJ, Lappi V, Wolfgang WJ, Lapierre P, Palumbo MJ, Medus C, Boxrud D 2015 Characterization of foodborne outbreaks of Salmonella enterica serovar Enteritidis with whole-genome sequencing single nucleotide polymorphism-based analysis for surveillance and outbreak detection. J Clin Microbiol 53:3334–3340. doi:10.1128/JCM.01280-15. 26269623 PMC4572550

[13] Kohl TA, Diel R, Harmsen D, Rothganger J, Walter KM, Merker M, Weniger T, Niemann S 2014 Whole-genome-based Mycobacterium tuberculosis surveillance: a standardized, portable, and expandable approach. J Clin Microbiol 52:2479–2486. doi:10.1128/JCM.00567-14. 24789177 PMC4097744

[14] Leopold SR, Goering RV, Witten A, Harmsen D, Mellmann A 2014 Bacterial whole-genome sequencing revisited: portable, scalable, and standardized analysis for typing and detection of virulence and antibiotic resistance genes. J Clin Microbiol 52:2365–2370. doi:10.1128/JCM.00262-14. 24759713 PMC4097726

[15] Ruppitsch W, Pietzka A, Prior K, Bletz S, Fernandez HL, Allerberger F, Harmsen D, Mellmann A 2015 Defining and evaluating a core genome multilocus sequence typing scheme for whole-genome sequence-based typing of Listeria monocytogenes. J Clin Microbiol 53:2869–2876. doi:10.1128/JCM.01193-15. 26135865 PMC4540939

[16] Maiden MC, Harrison OB 2016 Population and functional genomics of the Neisseria revealed with gene-by-gene approaches. J Clin Microbiol 54:1949–1955. doi:10.1128/JCM.00301-16. 27098959 PMC4963508

[17] Haendiges J, Jones J, Myers RA, Mitchell CS, Butler E, Toro M, Gonzalez-Escalona N 2016 A nonautochthonous U.S. strain of Vibrio parahaemolyticus isolated from Chesapeake bay oysters caused the outbreak in Maryland in 2010. Appl Environ Microbiol 82:3208–3216. doi:10.1128/AEM.00096-16. 26994080 PMC4959243

[18] Thomson NR, Clayton DJ, Windhorst D, Vernikos G, Davidson S, Churcher C, Quail MA, Stevens M, Jones MA, Watson M, Barron A, Layton A, Pickard D, Kingsley RA, Bignell A, Clark L, Harris B, Ormond D, Abdellah Z, Brooks K, Cherevach I, Chillingworth T, Woodward J, Norberczak H, Lord A, Arrowsmith C, Jagels K, Moule S, Mungall K, Sanders M, Whitehead S, Chabalgoity JA, Maskell D, Humphrey T, Roberts M, Barrow PA, Dougan G, Parkhill J 2008 Comparative genome analysis of Salmonella Enteritidis PT4 and Salmonella Gallinarum 287/91 provides insights into evolutionary and host adaptation pathways. Genome Res 18:1624–1637. doi:10.1101/gr.077404.108. 18583645 PMC2556274

[19] Kimura M 1980 A simple method for estimating evolutionary rates of base substitutions through comparative studies of nucleotide sequences. J Mol Evol 16:111–120. doi:10.1007/BF01731581. 7463489

[20] Tamura K, Stecher G, Peterson D, Filipski A, Kumar S 2013 MEGA6: Molecular Evolutionary Genetics Analysis version 6.0. Mol Biol Evol 30:2725–2729. doi:10.1093/molbev/mst197. 24132122 PMC3840312

[21] Clinical and Laboratory Standards Institute. 2016 Performance standards for antimicrobial susceptibility testing. Clinical and Laboratory Standards Institute, Wayne, PA.

[22] Chai SJ, White PL, Lathrop SL, Solghan SM, Medus C, McGlinchey BM, Tobin-D'Angelo M, Marcus R, Mahon BE 2012 Salmonella enterica serotype Enteritidis: increasing incidence of domestically acquired infections. Clin Infect Dis 54(Suppl 5):S488–S497. doi:10.1093/cid/cis231. 22572674

[23] Smith AM, Ismail H, Henton MM, Keddy KH 2014 Similarities between Salmonella Enteritidis isolated from humans and captive wild animals in South Africa. J Infect Dev Ctries 8:1615–1619. doi:10.3855/jidc.5393. 25500660

[24] Bell RL, Zheng J, Burrows E, Allard S, Wang CY, Keys CE, Melka DC, Strain E, Luo Y, Allard MW, Rideout S, Brown EW 2015 Ecological prevalence, genetic diversity, and epidemiological aspects of Salmonella isolated from tomato agricultural regions of the Virginia Eastern Shore. Front Microbiol 6:415. doi:10.3389/fmicb.2015.00415. 25999938 PMC4423467

[25] Blanc-Potard AB, Groisman EA 1997 The Salmonella selC locus contains a pathogenicity island mediating intramacrophage survival. EMBO J 16:5376–5385. doi:10.1093/emboj/16.17.5376. 9311997 PMC1170169

[26] Toft C, Andersson SG 2010 Evolutionary microbial genomics: insights into bacterial host adaptation. Nat Rev Genet 11:465–475. doi:10.1038/nrg2798. 20517341

[27] Retamal P, Castillo-Ruiz M, Villagra NA, Morgado J, Mora GC 2010 Modified intracellular-associated phenotypes in a recombinant Salmonella Typhi expressing *S*. Typhimurium SPI-3 sequences. PLoS One 5:e9394. doi:10.1371/journal.pone.0009394. 20195364 PMC2827545

[28] Achtman M, Wain J, Weill FX, Nair S, Zhou Z, Sangal V, Krauland MG, Hale JL, Harbottle H, Uesbeck A, Dougan G, Harrison LH, Brisse S 2012 Multilocus sequence typing as a replacement for serotyping in Salmonella enterica. PLoS Pathog 8:e1002776. doi:10.1371/journal.ppat.1002776. 22737074 PMC3380943

[29] Fernandez J, Fica A, Ebensperger G, Calfullan H, Prat S, Fernandez A, Alexandre M, Heitmann I 2003 Analysis of molecular epidemiology of Chilean Salmonella enterica serotype Enteritidis isolates by pulsed-field gel electrophoresis and bacteriophage typing. J Clin Microbiol 41:1617–1622. doi:10.1128/JCM.41.4.1617-1622.2003. 12682153 PMC153903

[30] Rios R, Araya R, Fernandez R, Tognarelli J, Hormazabal JC, Fernandez JO 2009 Subtipificacion molecular de Salmonella enterica serotipo Enteritidis en el periodo post epidemico. Rev Med Chile 137:71–75. 19399324

[31] Mathieu C, Moreno V, Pedersen J, Jeria J, Agredo M, Gutierrez C, Garcia A, Vasquez M, Avalos P, Retamal P 2015 Avian influenza in wild birds from Chile, 2007–2009. Virus Res 199:42–45. doi:10.1016/j.virusres.2015.01.008. 25602438

[32] Tran T, Andres P, Petroni A, Soler-Bistue A, Albornoz E, Zorreguieta A, Reyes-Lamothe R, Sherratt DJ, Corso A, Tolmasky ME 2012 Small plasmids harboring *qnrB19*: a model for plasmid evolution mediated by site-specific recombination at *oriT* and Xer sites. Antimicrob Agents Chemother 56:1821–1827. doi:10.1128/AAC.06036-11. 22290975 PMC3318318

[33] Wu S, Dalsgaard A, Hammerum AM, Porsbo LJ, Jensen LB 2010 Prevalence and characterization of plasmids carrying sulfonamide resistance genes among Escherichia coli from pigs, pig carcasses and human. Acta Vet Scand 52:47. doi:10.1186/1751-0147-52-47. 20670455 PMC2922292

[34] Pan American Health Organization. 2010 Informe anual de la Red Latinoamericana de Vigilancia de la Resistencia a los Antibióticos. Pan American Health Organization, Washington, DC http://www.paho.org/hq/index.php?option=com_docman&task=doc_view&gid=24101&Itemid=270.

[35] Ruppitsch W, Pietzka A, Prior K, Bletz S, Fernandez HL, Allerberger F, Harmsen D, Mellmann A 2015 Defining and evaluating a core genome multilocus sequence typing scheme for whole-genome sequence-based typing of Listeria monocytogenes. J Clin Microbiol 53:2869–2876. doi:10.1128/JCM.01193-15. 26135865 PMC4540939

