# Supplementary material for: Whole-Genome Sequencing Analysis of Salmonella enterica Serovar Enteritidis Isolates in Chile Provides Insights into Possible Transmission between Gulls, Poultry, and Humans
Source: Appl Environ Microbiol. 2016 Sep 30;82(20):6223–32. doi: 10.1128/AEM.01760-16 (PMC5068155; doi:10.1128/AEM.01760-16)
Supplement: Supplemental material [file supp_82_20_6223__index.html]

Supplemental material 

# Whole-Genome Sequencing Analysis of Salmonella enterica Serovar Enteritidis Isolates in Chile Provides Insights into Possible Transmission between Gulls, Poultry, and Humans

## Supplemental material

- Supplemental file 1 -

  Whole-genome phylogenetic analysis of *Salmonella* Enteritidis sequences available in GenBank (*n* = 274) and Chilean SE strains (*n* = 30), based on cgMLST results (Fig. S1).

  PDF, 3.2M
- Supplemental file 2 -

  List of SE genomes at GenBank used for comparison with Chilean SE genomes (Table S1).

  XLSX, 25K
- Supplemental file 3 -

  Core genes (loci) used for the analysis and their respective allele numbers for each individual Chilean SE strain analyzed in this study (Table S2).

  XLS, 2.1M
- Supplemental file 4 -

  Alleles found for each core gene (locus) shared by all Chilean SE strains analyzed in this study (Table S3).

  XLS, 149K
- Supplemental file 5 -

  SNPs found for each core locus shared by Chilean SE strains analyzed in this study (Table S4).

  XLS, 173K
- Supplemental file 6 -

  Alleles found for each core gene (locus) shared by all SE strains retrieved from NCBI and analyzed in this study (Table S5).

  XLSX, 999K
- Supplemental file 7 -

  SNPs found for each core locus shared by SE strains retrieved from NCBI and analyzed by cgMLST in this study (Table S6).

  XLSX, 638K
