# Supplementary material for: Whole-Genome Sequencing Analysis of Salmonella enterica Serovar Enteritidis Isolates in Chile Provides Insights into Possible Transmission between Gulls, Poultry, and Humans
Source: Appl Environ Microbiol. 2016 Sep 30;82(20):6223–32. doi: 10.1128/AEM.01760-16 (PMC5068155; doi:10.1128/AEM.01760-16)
Supplement: Supplemental material [file AEM.01760-16_zam999117464so1.pdf]

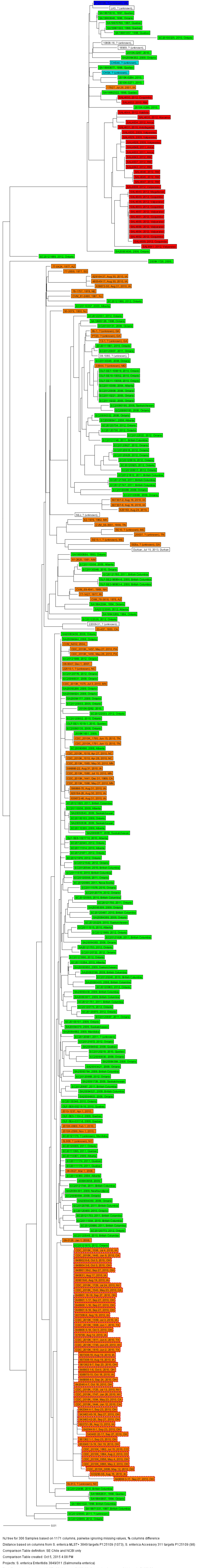

NJ tree for 306 Samples based on 1171 columns, pairwise ignoring missing values, % columns difference

Distance based on columns from S. enterica MLST+ 3849 targets P125109 (1073), S. enterica Accessory 311 targets P125109 (98)

Comparison Table definition: SE Chile and NCBI only

Comparison Table created: Oct 5, 2015 4:08 PM

Projects: S. enterica Enteritidis 3849/311 (Salmonella enterica)

Task Templates: S. enterica MLST+ 3849 targets P125109, S. enterica Accessory 311 targets P125109

- Chile SE
- USA
- Canada
- China
- Refe: UK
